# Assessing the Effectiveness of Providing Live Black Soldier Fly Larvae (*Hermetia illucens*) to Ease the Weaning Transition of Piglets

**DOI:** 10.3389/fvets.2022.838018

**Published:** 2022-02-16

**Authors:** Allyson F. Ipema, Walter J. J. Gerrits, Eddie A. M. Bokkers, Manon A. van Marwijk, Bjorge F. A. Laurenssen, Bas Kemp, J. Elizabeth Bolhuis

**Affiliations:** ^1^Adaptation Physiology Group, Department of Animal Sciences, Wageningen University and Research, Wageningen, Netherlands; ^2^Animal Nutrition Group, Department of Animal Sciences, Wageningen University and Research, Wageningen, Netherlands; ^3^Animal Production Systems Group, Department of Animal Sciences, Wageningen University and Research, Wageningen, Netherlands

**Keywords:** piglet, black soldier fly larvae (BSFL), enrichment, weaning, behavior, health, affective state, performance

## Abstract

Weaning is a stressful event for piglets, involving substantial changes to their nutritional and social environment. Providing edible enrichment around weaning may ease the weaning transition by increasing pre-weaning feed intake and improving post-weaning performance, health, behavior, and affective state. In this study, we investigated the effects of providing live black soldier fly larvae (BSFL) as edible enrichment pre- and/or post-weaning. Pre-weaning, piglets received either only creep feed (Pre-C, *n* = 14 litters) or creep feed and live BSFL (Pre-L, *n* = 15 litters) *ad libitum*, and post-weaning piglets either had no access to live BSFL (Post-C, *n* = 24 pens) or they could rotate tubes that released BSFL (Post-L, *n* = 24 pens) at levels up to 20% of their expected daily dry matter intake, resulting in treatments CC, CL, LC, and LL. No interaction between pre- and post-weaning treatment was found for any of the measured parameters. Before weaning, Pre-L piglets preferred to interact with larvae over creep feed, and Pre-C piglets interacted more with creep feed than Pre-L piglets. Total time spent on feed-directed behaviors did not differ. Continuous larvae provisioning increased caecum length and proximal stomach digesta pH, while it decreased the passage of glucose and fluorescein isothiocyanate through the colon wall on d3 post-weaning (CC vs. LL, *n* = 12 piglets/treatment). Post-weaning diarrhea and final body weight were not affected by treatment. After weaning, Pre-C piglets tended to eat more and grew marginally faster than Pre-L piglets. Post-C piglets spent more time eating and had a higher feed intake post-weaning than Post-L piglets. Based on home-pen behavioral observations, Post-L piglets actively explored and ate the larvae. Post-C piglets spent more time on exploring the environment and nosing pen mates, and they spent more time on manipulating pen mates on d8 and played more on d8 & 15 compared to Post-L piglets. Piglet responses to a novel environment and an attention bias test on d4 & 5 post-weaning were not influenced by larvae provisioning. In conclusion, pre-weaning larvae provisioning did not improve pre-weaning feed intake and post-weaning performance, however post-weaning larvae provisioning did benefit piglet behavior as less manipulation of pen mates was observed.

## Introduction

Weaning is a critical period for commercially housed piglets. Under natural conditions weaning is a gradual process lasting several months (1, 2), whereas commercial weaning often takes place abruptly when piglets are 3–4 weeks old. As a result all weaning-related stressors, including separation from the sow, switching from a milk to a concentrate diet, relocation to a new environment, and often mixing with unfamiliar conspecifics, occur acutely and simultaneously, intensifying the stress experienced by newly weaned piglets (3, 4). At this young age, piglets tend to have little to no experience with eating solid (creep) feed, and pre-weaning feed intake varies markedly between piglets and litters (5–8). Inexperience with solid feed combined with the stress of early weaning often causes a drop in feed intake and growth directly after weaning (9, 10). This, in turn, can compromise the development of the gastro-intestinal tract (GIT), resulting in, among others, villous atrophy and increased intestinal permeability (11–13). Suboptimal GIT functioning poses a threat to piglet performance and health, demonstrated by a high incidence of post-weaning diarrhea (14, 15). In addition to facing nutritional challenges, newly weaned piglets are often confronted with social and environmental challenges after weaning. Mixing of unacquainted piglets at weaning causes aggressiveness and fighting (16, 17), especially when piglets are housed in barren environments (18). Furthermore, pigs are highly motivated to perform foraging behaviors such as rooting (19), and when barren environments do not facilitate this behavior, it is often redirected to pen fixtures, or to other pigs in the form of damaging oral manipulations such as ear and tail biting (20, 21). The combined challenges of early and abrupt weaning thus diminish the welfare of newly weaned pigs and can have long-term ramifications for pig performance and health (13, 22).

A potentially effective strategy for easing the weaning transition is providing edible environmental enrichment pre- and/or post-weaning. Compared to providing only creep feed, providing additional edible items pre-weaning can create a more diverse diet in terms of texture, taste, smell, and nutrients. Previous studies found that providing a diet composed of several feed types before weaning enhances pre-weaning feed exploration and feed intake (23–25), and it can increase the number of piglets that sample solid feed before weaning (23). The increased interest in and consumption of a diverse diet likely occurs due to a decrease in sensory-specific satiety (26) and increased opportunities for exploration (23). Sufficient experience with eating feed before weaning can have post-weaning benefits such as improved performance (6, 27, 28), enhanced nutrient absorption in the small intestine (29), a more matured intestinal microbiota, and increased weight of several GIT segments (30). Furthermore, providing edible enrichment such as straw before weaning has been shown to attenuate stress responses toward humans and during transport at weaning (31). This indicates that piglets exposed to such enrichment may be better able to cope with the weaning transition.

Post-weaning, environmental enrichment such as increased space and/or access to straw or peat has been found to increase feed intake (32) and growth (33), and it can facilitate exploration and decrease damaging behaviors such as fighting and pig-directed oral manipulation (18, 33, 34). Enrichment can also benefit the affective state of pigs. Providing piglets with a combination of space, straw, and manipulatable objects caused a more positive affective state compared to barren-housed piglets (35). Similarly, having access to a wooden box with popcorn and wood shavings and/or an object made of plastic tubing caused fewer fear-related behaviors to be exhibited during social isolation (21). Edible enrichment items are expected to maintain interest longer than non-edible enrichment due to the positive reinforcement of consumption (36, 37).

A type of feed that is highly appropriate to be used as edible enrichment for pigs during the weaning transition is live black soldier fly larvae (*Hermetia illucens*, BSFL). These larvae are high in moisture, fat, and protein and low in carbohydrates (38–40). This makes them very palatable, and it makes them a suitable transition feed in the weaning period, because piglets are accustomed to liquid milk diets and digestion of larvae does not require starch-degrading enzymes that are uncommon in young piglets. Young piglets actively consumed live BSFL in previous studies (41, 42). Some observed benefits of BSFL inclusion (in different amounts of full-fat or defatted meal) in weaner pig diets are increased growth, increased beneficial bacteria in the gut, and increased villus height in the jejunum (43–45). In other cases, however, BSFL meal inclusion did not affect weaner pig performance (43, 46). In our previous study, piglets that were provided with small amounts of live BSFL for 11 days after weaning showed high levels of larvae-directed exploration, decreased levels of pig-directed oral manipulation and fighting, and decreased neophobic responses toward an unfamiliar object (41).

Taken together, we hypothesize that providing live BSFL around weaning has the potential to ease the weaning transition. In this study we therefore investigated the effects of larvae provisioning pre- and/or post-weaning on piglet performance, GIT development, health, behavior, and affective state. We expected that larvae provisioning before weaning would increase pre-weaning feed intake and benefit GIT development, and thereby improve post-weaning performance and health. We also expected that larvae provisioning during 3 weeks after weaning would benefit piglet feed intake, reduce the occurrence of maladaptive behaviors, and benefit the piglets' affective state. Keeping the type of feed or the environment similar during the pre- and post-weaning period is known to benefit, respectively, piglet feed intake (47) and behavior (18, 20, 48) after weaning, and reducing the amount of change during weaning can reduce weaning stress (3, 4). Therefore, piglets with continuous access to live BSFL around weaning were expected to experience the greatest benefits of larvae provisioning.

## Methods

The Animal Care and Use committee of Wageningen University & Research (Wageningen, The Netherlands) approved the applied protocols under project license number AVD1040020187184. The protocols were in accordance with the Dutch animal experimentation law that complies with European Directive 2010/63/EU. The inclusion of indigo carmine in creep feed was approved by the Medicines Evaluation Board (Utrecht, The Netherlands).

### Animals, Housing, and Management

In this study a 2 x 2 factorial design was applied, where piglets either had no access to larvae (Control, C) or had access to larvae (Larvae, L) pre-weaning (Pre-C and Pre-L) and/or post-weaning (Post-C and Post-L, resulting in the four treatment combinations CC, CL, LC, and LL, explained below). Twenty-nine multiparous pregnant sows (TN70 sows inseminated by Tempo boar semen, Topigs Norsvin, Vught, The Netherlands) were divided over two farrowing rooms in two successive batches (balanced for treatment), and they were assigned to treatments based on sow parity and piglets' birthdate (Pre-C: *n* = 14 sows, parity 4.7 ± 0.7, Pre-L: *n* = 15 sows, parity 4.6 ± 0.6). Approximately 2 weeks before farrowing the sows were transported from a conventional farm to the research facility of Wageningen University & Research. Here, sows were housed in groups of four or five familiar sows until 1 week before farrowing, and subsequently they were individually housed in farrowing pens until weaning. Sows received commercial gestation and lactation feeds (ForFarmers, Lochem, The Netherlands) in weighed portions at 7:30 and 16:00 h. To prevent piglets from consuming sow feed, any leftovers were removed 30 min after feed provisioning.

**Figure 1:**
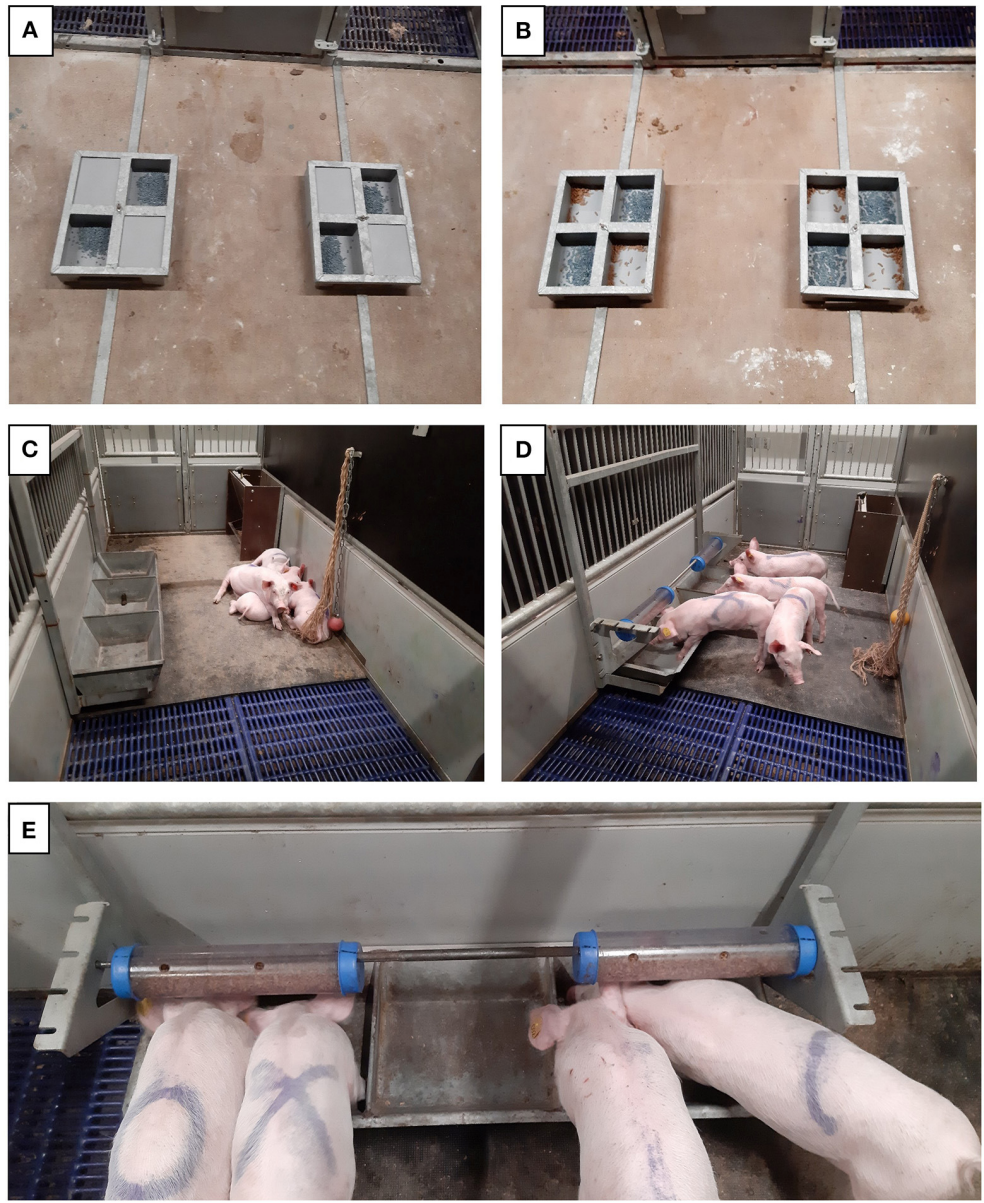
Set-up of the pre- and post-weaning treatments. From d3 pre-weaning, piglets could access the feeding area that contained either two feeders with two feeding spaces containing creep feed **(A)**, or two feeders with four feeding spaces, two containing creep feed and two containing larvae **(B)**. Post-weaning, piglets either had access to an empty feeder **(C)**, or access to a feeder to which two horizontally suspended tubes containing larvae were attached **(D)**. A close-up of the tubes filled with larvae can be seen in **(E)**.

The farrowing pen had a section with slatted flooring (2.85 x 1.80 m) containing the sow crate (2.85 x 0.6 m), and an adjacent piglet feeding area with concrete flooring (1.30 x 1.80 m). A rubber mat was placed under the sow and in the piglet nest for comfort. One heating lamp was placed at either side of the sow, and the height of the lamps was adjusted over the pre-weaning period. The sow crate contained a feed trough, drinking nipple and a chew object (a chain with either a rubber ball, several bolts, or a plastic ring with protrusions attached to it) that was changed every 3 days. Around farrowing one jute sack was available to the sow. Piglets had access to a drinking nipple and a chain with bolts attached to it in the slatted area. From d3 after birth, piglets could access the feeding area that contained two feeders with either two (Pre-C treatment) or four (Pre-L treatment) feeding bowls (17.5 x 13.5 cm per bowl, Figure 1). Room temperature at farrowing was 25°C, and this was decreased gradually to 21°C on d13, after which it remained constant.

Within 24 h after birth, piglets were weighed, and they received an ear tag and a 1 cc intramuscular iron injection. No castration, tail docking or teeth clipping were done. Within 2 days of age litter size was standardized by cross-fostering. At weaning on d28, litter size (Pre-C: 13.7 ± 0.3, Pre-L: 13.2 ± 0.3 piglets/litter) and weaning age (Pre-C: 26.4 ± 0.4, Pre-L: 27.1 ± 0.4 days old) did not differ significantly between treatments. At weaning, a subset of 240 piglets were transported to two weaner rooms in the two successive batches. Piglets were selected on health (no leg problems), sex, and body weight on d21 (close to average weight of the treatment group and the litter). Post-weaning, piglets were initially housed in groups of five piglets from five different litters to simulate commercial mixing at weaning. Piglets were housed in either a two female/three male or three female/two male ratio, and pens were assigned to one of the four treatment combinations (*n* = 12 pens per treatment combination), balanced per room. Three days after weaning, one piglet/pen was removed, and the removed piglets from the CC and LL treatments were sacrificed for post-mortem gastro-intestinal tract analysis (see below). From d3 to 21 post-weaning (the end of the experiment), all pens had a two female/two male ratio.

Pens in the weaner rooms (2.85 x 1.20 m) had half slatted and half rubber flooring and were equipped with a feed trough (12 x 50 cm with three feeding places), drinking nipple, hanging jute rope, and a chew object (a chain with either a rubber ball or a plastic ring with protrusions attached to it) that was changed weekly. All pens also contained an experimental feeder (1.0 x 0.3 m), and in the Post-L pens two transparent tubes (32 cm long, 7.5 cm Ø) with four 1 cm Ø holes at the top were horizontally suspended above the feeder at approximately piglet shoulder height (Figure 1). Piglets had *ad libitum* access to water and to a commercial pelleted weaner feed (Vida Prima 3, ForFarmers, Lochem, The Netherlands). At weaning, the room temperature was 25°C and this was gradually decreased to 23°C at d10, after which it remained constant.

In both the farrowing and weaner rooms the light and a radio were on from 07:00 to 19:00 h, and the lights were dimmed and the radio was off from 19:00 to 07:00 h.

### Experimental Design

Piglets were assigned to one of four treatment combinations in a 2 x 2 factorial design, where piglets did or did not receive larvae pre-weaning and/or for 3 weeks post-weaning. Live, 14-day-old black soldier fly larvae (BSFL) were provided weekly (by Bestico B.V., Berkel en Rodenrijs, The Netherlands) and they were stored at 12 °C until provisioning. The creep feed (Supplementary Tables 1, 2) provided pre-weaning was pelleted by Research Diet Services (Wijk bij Duurstede, The Netherlands) and contained 5 g/kg Indigo carmine feed colorant (E132 Eurocert 311811, Pomona Aroma, Hedel, The Netherlands) that turned the feed blue and allowed for visual identification of creep feed consumption in the feces.

#### Pre-weaning Treatments

In the farrowing pens, piglets had unrestricted access to the feeding area containing two feeders from d3 after birth. In pens in the Control (Pre-C) treatment each feeder had two diagonally adjacent feed bowls containing creep feed, and in pens in the Larvae (Pre-L) treatment each feeder had four adjacent feed bowls, two containing creep feed and two containing live BSFL (Figure 1). Creep feed was always provided in portions of approximately 25 g, and larvae were provided in portions of 25 g until d22, and from d22 to 28 they were provided in portions of 150 g to accommodate the increasing demand. Every morning, one portion of the appropriate feed item was added to each feed bowl to keep the feed fresh. Feed bowls were checked at least four times a day, and a portion was added to a feed bowl if it was almost empty, to ensure near *ad libitum* access to the feed items while minimizing leftovers. Once a week the feed bowls were cleaned, and the feed items were completely refreshed.

#### Post-weaning Treatments

After weaning, piglets were assigned either to the Control (Post-C) treatment that did not receive larvae, or to the Larvae (Post-L) treatment that received larvae in horizontally suspended tubes above a feeder (Figure 1), resulting in treatment combinations CC, CL, LC, and LL. Post-L piglets had to root or push the tubes to turn them at least 90° for the larvae to fall out. During the first 2 days after weaning, larvae were provided *ad libitum*, and on these days every morning and afternoon 420 g of larvae were placed in the feeder under the tubes to attract piglets to the tubes. After d2, piglets received up to 20% of their expected dry matter intake (based on manufacturer's recommendations) as live larvae in tubes, calculated per week. The maximum amount of live larvae provided per piglet per day was 140 g in w1, 270 g in w2, and 380 g in w3 post-weaning. Every day at 08:00, 12:00, and 16:00 h the tubes were checked, and near-empty tubes were refilled if the pen had not yet received its maximum daily amount of larvae.

### Measurements

#### Identification of Creep Feed Eaters Based on Rectal Swabs Pre-weaning

Rectal swabs were taken from each piglet on d7, 14, 21, and 28 pre-weaning (during the weighing procedure, see below) to identify creep feed eaters based on the blue color of the creep feed that was visible in the feces. Piglets were scored as being an eater (blue colored swab) or a non-eater (no blue colored swab). The percentage of eaters per litter on each measuring day was calculated. The creep feed eater types of piglets for the total pre-weaning period were determined, where piglets were ranked as non, bad, moderate, or good creep feed eaters if they had, respectively, zero, one, two or three-four blue colored swabs [modified from Pluske et al. (6)].

#### Feed-Directed Behavior and Identification of Eaters Based on Behavior Pre-weaning

One day before behavioral observations pre-weaning, up to 14 piglets per pen were marked (stock marker spray) for individual identification. Feed-directed behavior in the home pen was scored on d8, 15, 22, and 27 (ethogram in Table 1). Each day, scoring was done by 3-min instantaneous scan sampling for seven 1-hour periods, starting at 08:00, 09:15, 10:30, 12:15, 14:00, 15:15, and 16:30 h. Two observers scored one room each, switching rooms every hour. Observations were performed on a tablet with the software Observer 3.3 (Noldus Information Technology B.V., Wageningen, The Netherlands). Before observations, the observers were trained, and inter-observer reliability was sufficient [Fleiss kappa > 0.8, (49)]. For both creep feed and total (creep feed and/or larvae) eaters the percentage of eaters per litter per day was calculated, and for the whole pre-weaning period piglets were ranked as non, bad, moderate, or good eaters if they had been observed eating at least once on, respectively, zero, one, two or three-four observation days.

**Table 1:** Ethogram of feed-directed behavior observed in the home pen pre-weaning.

**Behavior**	**Description**
Exploring feeder	Sniffing, touching (with snout) or rooting feeder
Exploring or playing with feed	Sniffing, touching (with snout) or rooting feed, rolling feed item over the floor, walking around the pen with feed item in mouth, shaking head while having feed item in mouth
Eating feed	Eating or chewing feed from the feeders or the floor

*Behaviors scored during the pre-weaning home-pen observations of pigs having access to either only creep feed (Pre-C) or creep feed and live black soldier fly larvae (Pre-L). For the Pre-L pigs, scoring included the type of feed item, either creep feed or larvae, that the behavior was directed to. Ethogram adjusted from Middelkoop et al. (23)*.

#### Performance

Piglets were individually weighed within 24 h after birth, and on d7, 14, 21, and 28 (day of weaning) pre-weaning, and d1, 2, 7, 14, and 21 post-weaning. Feed intake pre-weaning could not be determined as piglets regularly spilled feed and larvae, which then got mixed with feces. Post-weaning, leftover feed and larvae were weighed back on d1, 2, 7, 14, and 21 to determine feed and larvae intake at pen level.

#### Fecal Consistency Scores

Post-weaning, the fecal consistency at piglet level was scored daily at 09:00 h by two observers. Score 1 represented firm feces, score 2 soft but shaped feces, score 3 loose feces and score 4 water thin feces (50). Each piglet was given a score based on the fecal consistency visible around the anus, and the total number of days piglets had diarrhea (score 3 or 4) or watery diarrhea (score 4) post-weaning were determined.

#### Gastro-Intestinal Tract Development

On d3 post-weaning, one piglet/pen of the CC and LL treatments (*n* = 12/treatment, balanced for sex and coming from different litters) was sacrificed by sedation and subsequent lethal injection with Euthasol for post-mortem gastro-intestinal tract measurements. The selected LL piglets were of similar weight and were observed to eat larvae at least three times during the pre-weaning behavioral observation on d22. The CC piglets were selected to be near the average weight of the selected LL piglets. The length, empty weight and digesta weight of the stomach, small intestine, caecum, and colon were recorded. The stomach was divided into the proximal and distal part by tying the middle off with a tie-wrap, and the proximal and distal digesta were collected separately and their pH was measured separately. Colonic digesta pH was also measured. Furthermore, a 20-cm section from the jejunum (proximal of small intestine midpoint) and from the colon (at colon midpoint) were cleaned with water, stripped of muscle, carefully everted, filled with a Ringer-HEPES solution, and tied off on both ends with rubber bands. These everted gut sacs were suspended in a closed Erlenmeyer in a solution containing Ringer, HEPES, glucose (900 μg/ml), and the fluorescent markers fluorescein isothiocyanate and tetramethyl rhodamine isothiocyanate (FITC and TRITC, respectively, 30 μg/ml, Sigma-Aldrich co. LLC., Saint Louis, USA). Before use, this solution was kept in a jerrycan containing an oxygen pump for oxygenation. The Erlenmeyers were placed in a water bath with shaker at 39°C for 1 h. Then, the sacs were removed, and their full and empty weight were determined, as well as their width and length to calculate the sac surface. The sac content was collected in black Eppendorf tubes and stored at −20°C. Before analysis, the samples were thawed at room temperature. The glucose concentration was measured using the Glucose liquiUV mono kit (HUMAN Gesellschaft für Biochemica und Diagnostica mbH, Wiesbaden, Germany). FITC and TRITC concentrations were determined by spectrophotometry (FITC measured at 485 nm excitation and 530 nm emission, TRITC measured at 528 nm excitation and 590 nm emission), and sample fluorescence was compared to a standard curve to determine the marker concentrations. From this, the transport per everted sac surface area (μg or nm/cm^2^) was calculated.

#### Post-weaning Home Pen Behavior

Piglets were marked weekly (stock marker spray) for individual identification. On d8, 15, and 20 post-weaning home pen behavior was observed through 2-min scan sampling for seven one-hours periods a day, starting at 08:00, 09:15, 10:30, 12:15, 14:00, 15:15, and 16:30 h (ethogram in Table 2). As for the pre-weaning observations, two observers with sufficient inter-observer reliability [Fleiss kappa > 0.8, (49)] scored one room each, switching rooms every hour, and observations were done on a tablet with the software Observer 3.3.

**Table 2:** Ethogram of behavior observed in the home pen post-weaning.

**Behavior**	**Description**
**Ingestive behavior**	
Eating feed	Chewing or swallowing feed pellets
Eating larvae	Chewing or swallowing larvae
Drinking	Drinking from water nipple
**Postures and locomotion**	
Inactive	Sitting, or lying on side or belly, without performing any other behavior
Standing and Walking	Standing idle with four hooves on the floor or walking without performing any other behavior
**Exploratory behavior**	
Exploring environment	Sniffing, touching (with snout), rooting, or chewing the pen floor, wall, toy/rope, feeder, or water nipple, or chewing air or feces
Exploring feeder (including tubes)	Sniffing, touching (with snout), rooting, or chewing the tubes containing larvae (present in Post-L treatment) or the experimental feeder to which the tubes can be attached (present in Post-C and Post-L treatment)
**Pig-directed behavior**	
Nose-to-nose	Having nose to nose contact with a pen mate
Nosing pen mate	Sniffing or touching (with snout) body of pen mate except the snout, including anal nosing
Manipulating pen mate	Mounting pen mate or nibbling, sucking, rooting, or chewing any part of a pen mate, including belly nosing
Fighting	Mutual pushing, pressing, ramming, head knocking, nudging, aggressively biting, or lifting pen mate
**Other behaviors**	
Play	Running, jumping, or turning in the pen (either individually or with pen mates), shaking head while holding toy/rope, pulling on toy/rope
Comfort behavior	Rubbing body against wall/floor, scratching body with hind leg, or stretching (part of) body
Other	Any behavior not described

*Behaviors scored during the post-weaning home pen observations of piglets that had access to no larvae (Post-C) or had access to hanging tubes containing live black soldier fly larvae (Post-L)*.

#### Affective State

As coping style can affect a piglet's behavioral response in a novel environment test (51) and in an attention bias test (52), piglet coping style was assessed through a back-test [based on Bolhuis et al. (53)] on d16-17 pre-weaning. In short, piglets were individually transported in a closed cart to a quiet room near the farrowing room, where they were placed on their back on a soft surface and manually restrained for 60 s during which the number of struggles and vocalizations were recorded. In accordance with Melotti et al. (54) piglets were classified as low resisters if they struggled zero or one times, or if they struggled twice and vocalized <25 times. Piglets that struggled three times or more, or that struggled twice and vocalized 25 times or more were classified as high resisters.

To assess affective state after weaning a novel environment test (NET, d4 post-weaning) and an attention bias test (ABT, d5 post-weaning) were performed. Two piglets per pen (one female/one male) were included in both tests. For the NET, piglets were individually caught and transported in a closed cart to an experimental room near the weaner rooms. Here, they were placed in a start box alongside the test area. Within 15 s a door was opened, and the piglet entered an unfamiliar area with a 5.3 x 5.3 m rubber floor surrounded by 1 m high hardwood walls. In the center of the floor stood a feed bowl containing 0.5 kg of their regular feed mixed with 10 raisins and 10 pieces of corn. After entering the area, the piglet's behavior was recorded for 5 min by two observers, on a tablet with the Observer 3.3 software (ethogram in Table 3). One observer scored behavioral states and another scored behavioral events. After the test ended, the piglet was transported to the home pen, and the test area was cleared of feces and urine and cleaned with cleaning solution and a moist mop.

**Table 3:** Ethogram of behaviors observed during the novel environment test and the attention bias test.

**Behavior**	**Description**
**Attentive states^a^**	
Attention to the threat	Having head oriented toward the location of the threat
Attention not to the threat	Having head oriented away from the location of the threat
**Behavioral states**	
Standing alert	Standing motionless with head fixed (up or down) and ears upright
Moving	Walking or running without performing any other described behavior. All four legs move, or the pig turns around on the same spot without moving all four legs
Standing	Standing, not alert, with four hooves on the floor without performing any other described behavior
Sitting/lying	Sitting on the floor, or lying on side or belly, without performing any other described behavior
Exploring environment	Exploring the floor or wall by sniffing, touching (with snout), rooting, chewing, or licking it
Exploring feed bowl	Exploring the feed bowl by sniffing, touching (with snout), rooting, chewing, or licking it. Rooting disc can be in contact with feed bowl, but pig is not eating
Eating feed	Chewing or swallowing feed. The eating event continues while the pig is chewing, provided that the head stays close to the feed bowl and the pig remains non-vigilant. Once the pig becomes vigilant or moves away from the feed bowl, this is the end of eating, even if the pig continues chewing
**Behavioral events**	
Low-pitched vocalizations	Short or long grunts
High-pitched vocalizations	Grunt-squeals, squeals, or screams
Eliminating	Excreting urine or feces
Escape attempt	Jumping in air or against the wall of the area

a *Only observed during the attention bias test*.

For the ABT, two-thirds of the piglets (*n* = 16/treatment) were tested with a threat, and one-third of the piglets (*n* = 8/treatment) were tested without a threat to assess threat effectiveness. The experimental procedure of the ABT was similar as that of the NET, however the ABT lasted 3 min. The threat was a combination of a flashing light and a siren. Ten s after entering the test area a door in the right wall (in relation to the start box) opened, and the flashing light was shown, and the siren was turned on. After 10 s the door closed, and the flashing light and siren were turned off. In addition to scoring the behaviors that were also scored in the NET, one observer scored the attention of the piglets toward the threat (Table 3).

### Statistical Analysis

#### Data Processing

Pre- and post-weaning piglet behaviors in the home pen were averaged per piglet per day and expressed as the proportion of scans. Post-weaning, the behaviors “Drinking,” “Fighting,” “Nose-to-nose,” and “Comfort behavior” were excluded from analysis due to their low occurrence (<1.5% of observations). A factor analysis was conducted on behaviors performed in the NET, and on the behaviors performed during the 150 s following the threat in the ABT. The behaviors “Sitting/lying” and “Escape attempt” were rare and therefore excluded from analysis. The occurrence of “Eating feed” was very low and therefore it was included in “Exploring feed bowl,” and the latency of this behavior was also incorporated in the analysis. When piglets did not interact with the feed bowl, the latency was set to the maximum possible time. The distribution of “High-pitched vocalizations” was skewed, and this behavior was combined with “Low-pitched vocalizations” into “Vocalizing.” The behaviors “Standing” (NET and ABT after threat) and “Exploring feed bowl” (ABT after threat) were arcsine square root transformed, and “Eliminating” (NET) was squared for normalization.

#### Data Analysis

All analyses were performed with the statistical software SAS 9.4 (SAS Institute Inc., Cary, NC, USA). General linear (mixed) model residuals were checked for normality. Except for the models on larvae consumption and larvae-directed behavior, all models on pre-weaning data included pre-weaning treatment as fixed effect, and all models on post-weaning data included pre-weaning treatment, post-weaning treatment and their interaction as fixed effects. Additionally, all models included batch as fixed effect. If data was on piglet level and multiple piglets from the same pen were included, a random pen effected nested within batch and in pre-weaning treatment or pre- and post-weaning treatment was always included.

Piglet growth rate, body weight, feed intake and GIT measurements were analyzed in general linear (mixed) models (MIXED in SAS). For analysis of piglet weaning weight and the weight on d21 post-weaning birth weight was included as covariate. For models analyzing organ and digesta weights, piglet body weight on d2 post-weaning was included as covariate.

The proportion of creep feed eaters and total (creep feed and/or larvae) eaters per pen based on swabs and behavior, and the proportion of scans spent on pre-weaning and post-weaning home-pen behaviors were analyzed in generalized linear (mixed) models (GLIMMIX in SAS) with a binomial distribution, logit link function and an additional multiplicative overdispersion parameter. Pre-weaning home-pen behavior was analyzed per day, and post-weaning home-pen behavior was analyzed for the entire post-weaning period combined. As such, models on post-weaning behavior included a fixed effect of day and its two-way interactions with pre- and post-weaning treatment, a random pen by day effect, and a repeated effect of day with piglet as subject using a heterogenous first-order autoregressive covariance structure. Initial models included the three-way interaction between pre-weaning treatment, post-weaning treatment, and day, however as this never had a significant effect it was removed from the final models.

The proportion of observations Pre-L piglets spent on interacting with larvae vs. creep feed was analyzed in a GLIMMIX with binomial distribution, logit link function, and overdispersion parameter. This model included a fixed effect of feed type, and a repeated effect of feed type with piglet as subject, using a compound symmetry covariance structure. Pre-weaning creep feed and total eater types were analyzed in a GLIMMIX with a multinomial distribution and cumulative logit link function. Number of days with (watery) diarrhea was analyzed in a GLIMMIX with a Poisson distribution, log link function and an additional multiplicative overdispersion parameter.

To assess the effectiveness of the threat during the ABT, the behavior of pigs that did or did not receive a threat was assessed in models with threat (yes or no) as a fixed effect. The proportion of time spent on behaviors was analyzed in a GLIMMIX with a binomial distribution, logit link function, and an additional multiplicative overdispersion parameter, the frequencies of behaviors were analyzed in a GLIMMIX with a Poisson distribution, log link function, and an additional multiplicative overdispersion parameter, and the latency to explore the feed bowl was analyzed in a MIXED model. For piglets receiving the threat, similar models were used to analyze behavior during the 10 s threat, and the fixed effects in this model were pre-weaning treatment, post-weaning treatment, and their interaction. The behaviors “Standing” and “Eliminating” were rare during the 10 s threat and were therefore not analyzed. The variables from the NET and ABT after the threat were put in factor analyses with orthogonal Kaiser-Varimax rotation. For the NET, the behavior “Eliminating” had a communality estimate below 0.3, therefore this behavior was excluded from the factor analysis. Based on Kaiser's criterium, factors with an eigenvalue above one were retained, resulting in two factors for the NET and three factors for the ABT after the threat (Table 4). The scores of each piglet for each factor were analyzed with MIXED models which initially included an additional fixed effect of coping style, however as this effect was never significant it was removed from the final models.

**Table 4:** Loadings of the factors with an eigenvalue above one that were extracted by factor analysis with orthogonal Kaiser-Varimax rotation on the behaviors and attention scored during the novel environment test and the attention bias test during the 150 s after the threat.

**Variable**	**Novel environment test**		
	**Factor 1**	**Factor 2**	
Moving (% of time)	**0.90**	−0.14	
Standing (% of time)	0.11	**0.55**	
Standing alert (% of time)	0.09	**0.86**	
Exploring environment (% of time)	**−0.82**	−0.41	
Exploring feed bowl (% of time)	0.02	**−0.61**	
Latency exploring feed bowl (s)	**−0.47**	0.39	
Vocalizing (frequency)	**0.59**	0.17	
*Eigenvalues*	2.07	1.79	
*% of variance explained*	42.7%	31.6%	
	**Attention bias test after threat**		
	**Factor 1**	**Factor 2**	**Factor 3**
Attention to threat (% of time)	**−0.48**	−0.40	−0.08
Moving (% of time)	**0.88**	0.19	0.17
Standing (% of time)	−0.06	0.03	**0.83**
Standing alert (% of time)	**−0.59**	**−0.79**	−0.14
Exploring environment (% of time)	−0.09	**0.98**	−0.04
Exploring feed bowl (% of time)	**0.64**	0.09	−0.29
Latency exploring feed bowl (s)	**−0.75**	−0.05	0.28
Vocalizing (frequency)	**0.72**	−0.02	−0.03
Eliminating (frequency)	−0.06	0.03	**0.67**
*Eigenvalues*	2.86	1.79	1.35
*% of variance explained*	48.7%	23.3%	16.3%

*High loadings (≤ – 0.45 or ≥ 0.45) are indicated in bold*.

Data are presented as pen means ± SEM unless stated otherwise. *P*-values below 0.05 were considered significant, and *p*-values between 0.05 and 0.1 were considered a trend. Significant fixed effects were analyzed on *post-hoc* differences in least square means with a Tukey's HSD correction.

## Results

### Pre-weaning Eaters

**Figure 2:**
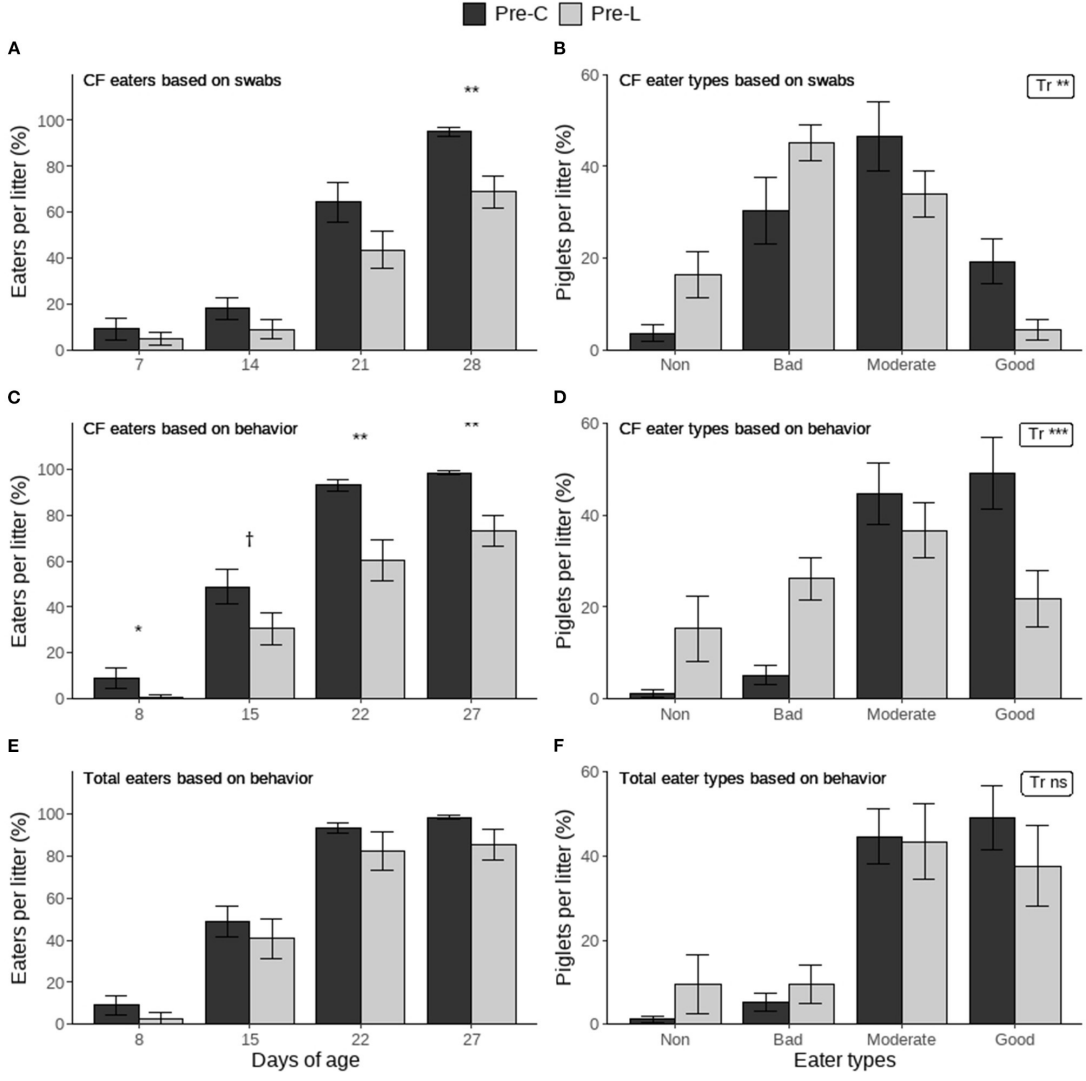
The percentage of piglets per litter that ate creep feed (CF) or creep feed and/or larvae (Total) per day and individual eater types determined at the end of the pre-weaning period based on blue colored rectal swabs or pre-weaning home pen behavioral observations. Piglets had access to either only creep feed (Pre-C) or creep feed and live black soldier fly larvae (Pre-L). For the percentage of eaters (per day) and for the eater types (distribution of all types) the treatment effect is indicated as ^†^(*p* < 0.1), ^*^(*p* < 0.05), ^**^(*p* < 0.01), ^***^(*p* < 0.001) or ns (not significant). Data are presented as pen means ± SEM.

The percentage of creep feed eaters per litter based on rectal swabs was significantly higher in the Pre-C treatment than in the Pre-L treatment at weaning but not on preceding days (Figure 2A). The distribution of creep feed eater types based on swabs was also affected by treatment, with the Pre-C treatment having more piglets in the better eater categories (Figure 2B). Similar results were found for the behavioral observations, where the percentage of creep feed eaters per litter was higher in the Pre-C treatment on d8, 22 and 27, and it tended to be higher on d15 (Figure 2C). Creep feed eater types based on behavior were also affected by treatment, with the Pre-C treatment again having more piglets in the better eater categories (Figure 2D). The percentage of total (creep feed and/or larvae) eaters per litter and the total eater types based on behavior were not influenced by treatment (Figures 2E,F).

### Pre-weaning Feed-Directed Behavior

**Figure 3:**
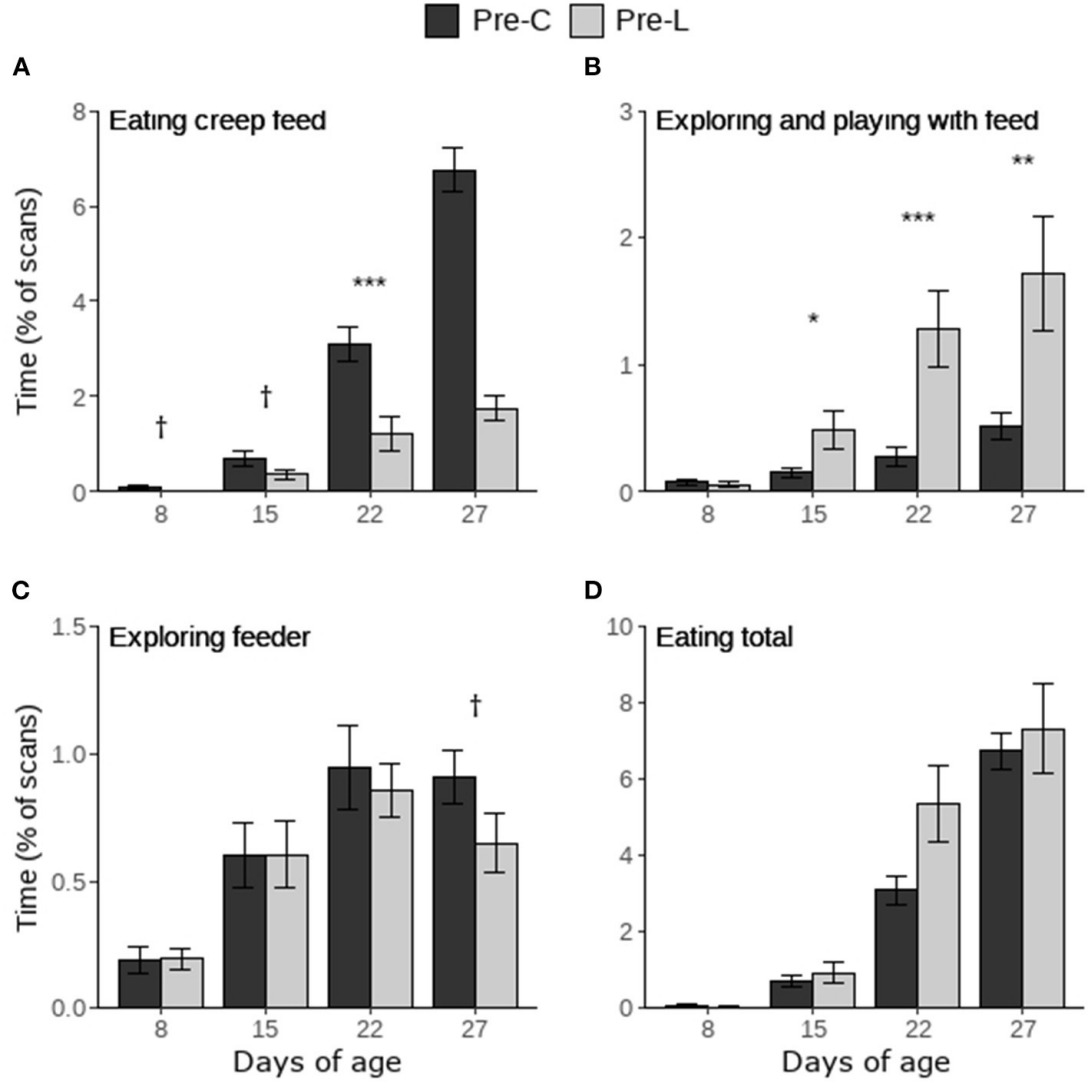
Time spent on feed-directed behaviors scored during the pre-weaning home pen observations of piglets having access to either only creep feed (Pre-C) or creep feed and live black soldier fly larvae (Pre-L). “Eating total” includes the time spent eating creep feed and eating larvae. Per day, treatment effects are indicated as ^†^(*p* < 0.1), ^*^(*p* < 0.05), ^**^(*p* < 0.01), or ^***^(*p* < 0.001). Data are presented as pen means ± SEM.

**Figure 4:**
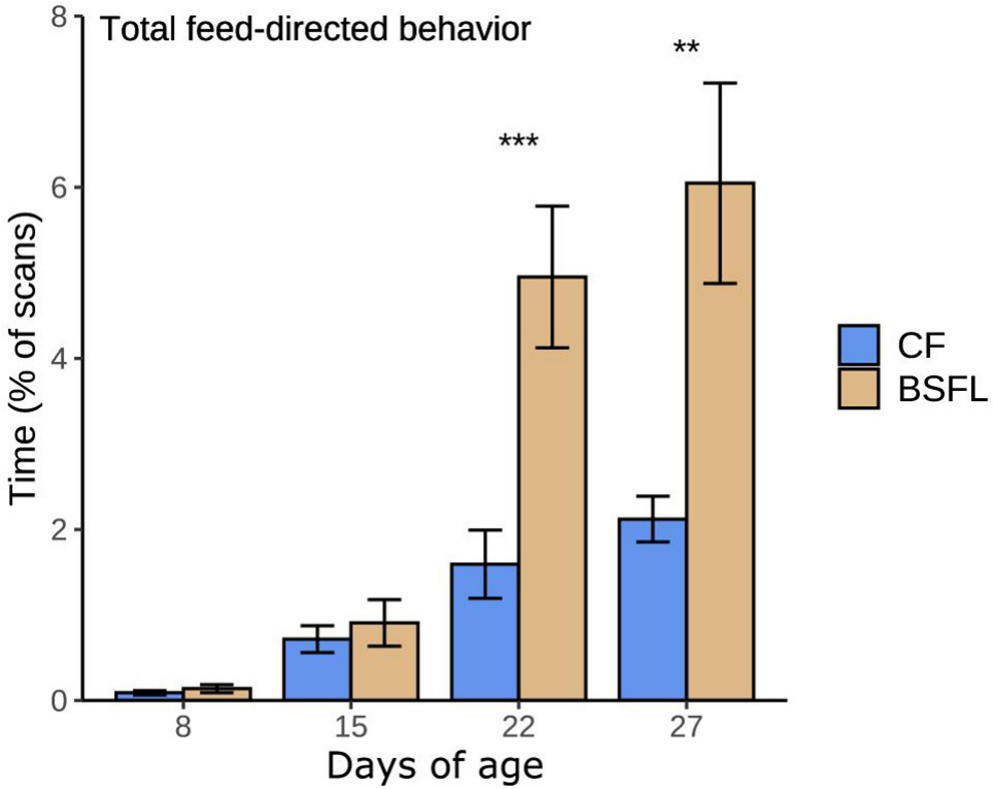
Time spent on all feed-directed behaviors (exploring feeder, exploring or playing with feed, and eating feed) toward creep feed (CF) and black soldier fly larvae (BSFL) as observed in the home pen of Pre-L piglets. Per day, effects of feed type are indicated as ^**^(*p* < 0.01) or ^***^(*p* < 0.001). Data are presented as pen means ± SEM.

Piglets in the Pre-C treatment tended to spend more time eating creep feed on d8 and 15, and they spent more time eating creep feed on d22 and 27 compared to Pre-L piglets (Figure 3A). On d8 treatment did not influence the time spent exploring and playing with the feed items, however Pre-L piglets spent more time exploring and playing with feed on d15, 22 and 27 compared to Pre-C piglets (Figure 3B). Only on d27 there was a trend for Pre-C piglets to spend more time exploring the feeder (Figure 3C). Treatment did not influence the total time spent eating (creep feed and/or larvae, Figure 3D). Within the Pre-L treatment, the time spent on interacting with creep feed or with larvae did not differ on d8 and 15, but piglets interacted more with larvae than with creep feed on d22 and 27 (Figure 4).

### Performance

#### Pre-weaning Performance

Pre-weaning, piglet birth weight, growth, and weaning weight were not affected by pre-weaning treatment (Table 5).

**Table 5:** Pre-weaning piglet average daily gain and body weight.

	**Pre-C**	**Pre-L**	***P*-value**
**ADG (g/piglet/day)**			
birth-d7	123 ± 9	125 ± 12	0.946
d7-14	217 ± 8	216 ± 13	0.913
d14-21	247 ± 9	258 ± 11	0.447
d21-28	293 ± 11	293 ± 12	0.911
Total, birth-d28	228 ± 7	229 ± 9	0.871
**Body weight (kg)**			
Birth	1.46 ± 0.03	1.49 ± 0.04	0.706
d28	7.56 ± 0.23	7.71 ± 0.28	0.604

*Pre-weaning average daily gain (ADG) and body weight of piglets having access to either only creep feed (Pre-C) or creep feed and live black soldier fly larvae (Pre-L). Data are presented as litter means ± SEM*.

#### Post-weaning Performance

No interaction was found between pre- and post-weaning treatment regarding any of the post-weaning performance parameters (Table 6).

**Table 6:** Post-weaning piglet average daily gain, feed consumption, and days with (watery) diarrhea.

	**Pre-C**		**Pre-L**		***P*** **-value**		
	**Post- C**	**Post-L**	**Post-C**	**Post-L**	**Pre**	**Post**	Pre^*^Post
**ADG (g/pig/day)**							
d0-1	93 ± 58	26 ± 31	−41 ± 42	6 ± 35	*0.073*	0.831	0.199
d1-2	284 ± 24	216 ± 23	315 ± 20	221 ± 22	0.402	**<0.001**	0.537
d2-7	149 ± 8	100 ± 17	168 ± 20	145 ± 20	*0.055*	**0.032**	0.408
d7-14	369 ± 15	340 ± 16	343 ± 20	324 ± 22	0.228	0.161	0.777
d14-21	563 ± 19	552 ± 24	482 ± 25	495 ± 33	**0.009**	0.981	0.626
Total d0-21	365 ± 10	333 ± 10	329 ± 16	316 ± 16	*0.060*	0.118	0.497
BW (kg) at d21	15.1 ± 0.2	14.5 ± 0.2	14.6 ± 0.4	14.5 ± 0.4	0.363	0.236	0.927
**DMI(g/pig/day) pellets**							
d0-1	183 ± 20	115 ± 12	148 ± 19	114 ± 15	0.226	**0.002**	0.268
d1-2	233 ± 17	115 ± 8	212 ± 15	117 ± 10	0.489	**<0.001**	0.371
d2-7	208 ± 9	139 ± 10	210 ± 11	142 ± 14	0.779	**<0.001**	0.949
d7-14	385 ± 13	245 ± 12	357 ± 24	235 ± 18	0.233	**<0.001**	0.600
d14-21	746 ± 27	484 ± 17	647 ± 30	459 ± 25	**0.013**	**<0.001**	0.125
Total d0-21	438 ± 14	281 ± 10	394 ± 16	271 ± 13	*0.059*	**<0.001**	0.239
**DMI(g/pig/day) BSFL**							
d0-1	-	54 ± 5	-	56 ± 6	0.802	-	-
d1-2	-	61 ± 3	-	66 ± 5	0.374	-	-
d2-7	-	28 ± 3	-	39 ± 3	**0.021**	-	-
d7-14	-	77 ± 4	-	86 ± 4	*0.057*	-	-
d14-21	-	136 ± 0	-	135 ± 0	0.339	-	-
Total d0-21	-	82 ± 1	-	88 ± 2	*0.056*	-	-
**DMI(g/pig/day) total**							
d0-1	183 ± 20	169 ± 13	148 ± 19	169 ± 18	0.243	0.781	0.243
d1-2	233 ± 17	176 ± 8	212 ± 15	183 ± 10	0.614	**0.001**	0.260
d2-7	208 ± 9	167 ± 10	210 ± 11	181 ± 13	0.427	**0.002**	0.569
d7-14	385 ± 13	323 ± 13	357 ± 24	321 ± 19	0.361	**0.002**	0.407
d14-21	746 ± 27	620 ± 17	647 ± 30	594 ± 26	**0.013**	**<0.001**	0.128
Total d0-21	438 ± 14	363 ± 10	394 ± 16	359 ± 14	*0.091*	**<0.001**	0.170
**Diarrhea**							
# of days with diarrhea	6.0 ± 0.8	6.2 ± 0.7	5.1 ± 0.6	6.0 ± 1.0	0.355	0.416	0.615
# of days with watery diarrhea	1.7 ± 0.4	1.6 ± 0.2	1.9 ± 0.3	2.0 ± 1.0	0.903	0.696	0.669

Post-weaning average daily gain (ADG), final body weight, dry matter intake (DMI) of pellets, BSFL, and both pellets and BSFL combined (total), and days with (watery) diarrhea of piglets that had access to either only creep feed (Pre-C) or creep feed and live black soldier fly larvae (Pre-L) pre-weaning, and consequently had access to no larvae (Post-C) or had access to live black soldier fly larvae (Post-L) post-weaning. P-values of the effect of pre-weaning treatment (Pre), post-weaning treatment (Post) and their interaction (Pre^*^Post) are presented, and significant effects (p < 0.05) are indicated in bold and trends (p < 0.1) are indicated in italic. Data are presented as pen means ± SEM.

Pre-weaning treatment influenced piglet growth (Table 6). Pre-C piglets tended to grow faster on the 1st day after weaning and in total from d0 to 21 after weaning, and they grew faster during d14-21 than Pre-L piglets. Conversely, during d2-7 Pre-L piglets tended to grow faster than Pre-C piglets. Post-weaning treatment also affected piglet growth (Table 6), as Post-C piglets grew faster during d1-2 and d2-7 than Post-L piglets.

Feed intake was influenced by pre-weaning treatment (Table 6). Pre-C piglets consumed more pellets during d14-21 post-weaning and tended to consume more pellets during the total post-weaning period from d0-21 than Pre-L piglets. Daily larvae dry matter consumption was higher for Pre-L piglets during d2-7, and it tended to be higher for Pre-L piglets during d7-14 and in total from d0-21 compared to Pre-C piglets. Pre-C piglets had a higher total daily dry matter intake (from pellets and larvae) during d14-21, and it tended to be higher in total from d0-21 compared to Pre-L piglets. Feed intake was also affected by post-weaning treatment (Table 6). Post-C piglets consumed more pellets than Post-L piglets during all periods. Post-C piglets also consumed more total dry matter than Post-L piglets during all periods except d0-d1.

The number of days with (watery) diarrhea and the body weight on d21 post-weaning were not affected by pre- and/or post-weaning treatment (Table 6).

### Gastro-Intestinal Tract Development

Post-mortem analysis indicated that the caecum of LL piglets was longer and tended to be heavier than the caecum of CC piglets (Table 7). Treatment did not affect the other segment lengths, empty weights and digesta weights. The proximal stomach digesta pH was higher for LL piglets than CC piglets, and the distal stomach and colon digesta pH did not differ between treatments. Compared to CC piglets, LL piglets had lower glucose and FITC passage through the colon wall, but not through the jejunum wall, and the TRITC passage through the jejunum and colon wall was not affected by treatment (Table 7).

**Table 7:** Post-mortem gastro-intestinal tract measures on d3 post-weaning.

**Variable**	**CC**	**LL**	***P*-value**
Body weight d2 (kg)	8.43 ± 0.13	8.43 ± 0.17	1.000
**Segment length**			
Small intestine (m)	10.6 ± 1.4	11.0 ± 1.0	0.399
Caecum (cm)	13.5 ± 4.1	16.2 ± 5.2	**0.046**
Colon (cm)	174.2 ± 31.4	176.6 ± 20.4	0.837
**Segment empty weight (g)**			
Stomach	61.6 ± 7.3	68.0 ± 14.2	0.178
Small intestine	299.7 ± 53.0	324.0 ± 54.0	0.268
Caecum	19.8 ± 4.0	23.2 ± 6.6	*0.080*
Colon	66.7 ± 14.7	69.9 ± 20.3	0.546
**Digesta weight (g)**			
Stomach	195.2 ± 79.4	181.2 ± 62.6	0.650
Small intestine	179.5 ± 27.5	147.9 ± 19.1	0.486
Caecum	47.9 ± 25.7	59.6 ± 16.3	0.205
Colon	109.7 ± 48.0	101.8 ± 38.5	0.653
**Digesta pH**			
Proximal stomach	3.96 ± 0.15	4.71 ± 0.29	**0.017**
Distal stomach	3.34 ± 0.24	3.10 ± 0.34	0.563
Colon	6.26 ± 0.06	6.29 ± 0.11	0.801
**Glucose passage (μg/cm**^**2**^ **sac surface)**			
Jejunum	10.7 ± 2.8	6.2 ± 1.4	0.164
Colon	4.9 ± 1.5	2.0 ± 0.8	**0.043**
**FITC passage (ng/cm**^**2**^ **sac surface)**			
Jejunum	88 ± 7	89 ± 9	0.955
Colon	163 ± 18	112 ± 14	**0.036**
**TRITC passage (ng/cm**^**2**^ **sac surface)**			
Jejunum	65 ± 4	71 ± 8	0.472
Colon	96 ± 11	77 ± 6	0.153

*Gastro-intestinal tract segment length, segment weight, digesta weight, digesta pH and marker passage through the everted intestinal sacs of piglets that had no access to larvae (CC) or pigs that had access to live black soldier fly larvae during the pre- and post-weaning period (LL). Significant effects (p < 0.05) are indicated in bold and trends (p < 0.1) are indicated in italic. Data are presented as pig means ± SEM*.

### Post-weaning Home Pen Behavior

**Figure 5:**
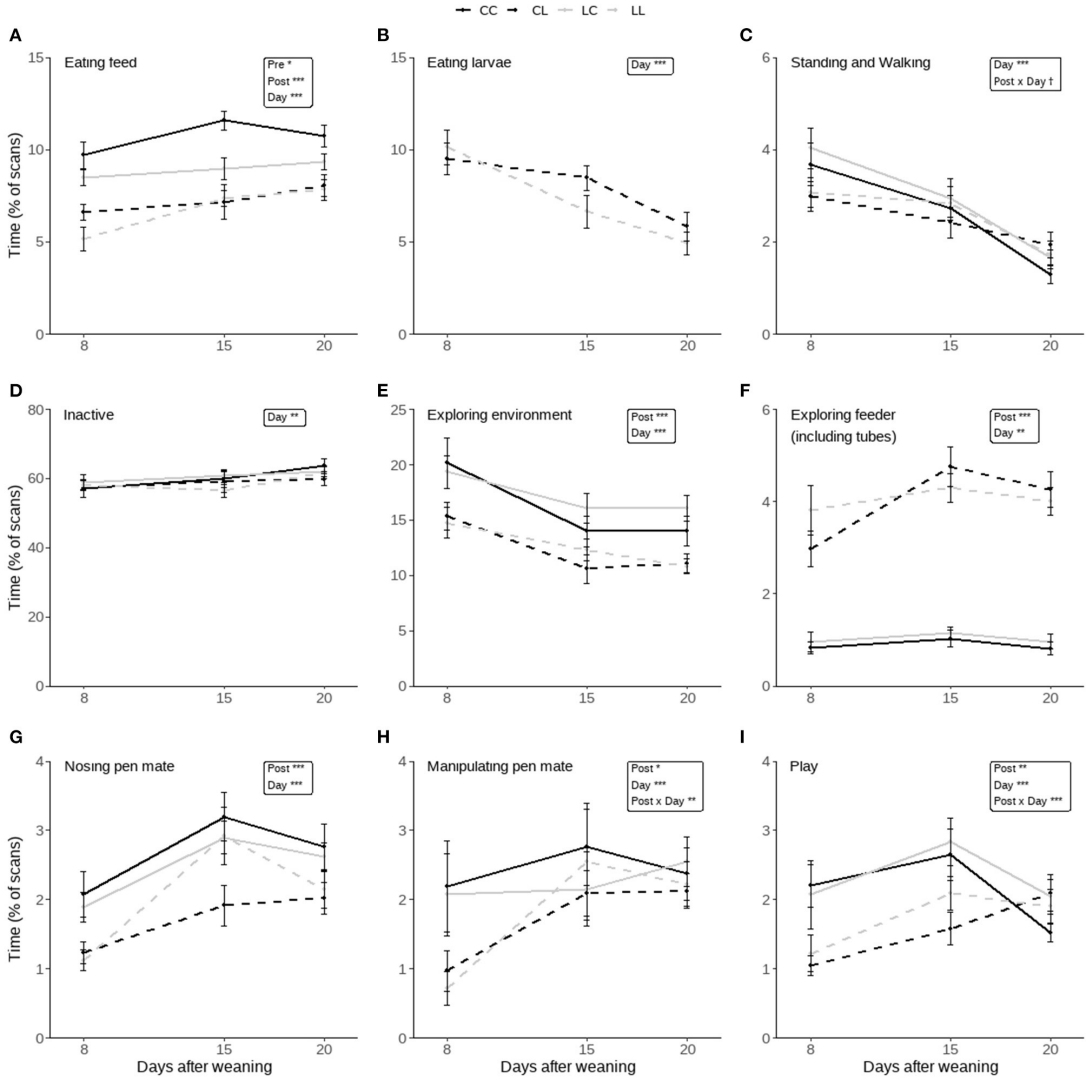
Time spent on behaviors scored during the post-weaning home pen observations of piglets that had access to either only creep feed (Pre-C) or creep feed and live black soldier fly larvae (Pre-L) pre-weaning, and consequently had access to no larvae (Post-C) or had access to live black soldier fly larvae (Post-L) post-weaning, resulting in treatments CC, CL, LC, and LL. Any effects of pre-weaning treatment, post-weaning treatment, day, and their 2-way interactions are indicated as ^†^(*p* < 0.1), ^*^(*p* < 0.05), ^**^(*p* < 0.01), or ^***^(*p* < 0.001), and non-significant (*p* > 0.1) effects are not indicated. Data are presented as pen means ± SEM.

The effects of pre-weaning treatment, post-weaning treatment, day, and their two-way interactions on the piglets' time spent on distinct home-pen behaviors post-weaning are shown in Figure 5. Significant effects are discussed below.

#### Ingestive Behavior

Pigs in the Pre-C treatment spent more time eating feed than those in the Pre-L treatment (Pre-C: 9.0 ± 0.3, Pre-L: 7.8 ± 0.3%), and pigs in the Post-C treatment spent more time eating feed than pigs in the Post-L treatment (Post-C: 9.8 ± 0.3, Post-L: 7.0 ± 0.3%). On d15 and 20 the time spent eating feed was higher than on d8 (d8: 7.5 ± 0.4, d15: 8.8 ± 0.4, d20: 9.0 ± 0.3%, Figure 5A). The time spent eating larvae decreased over time; it was higher on d8 than d15 and 20, and it was higher on d15 than on d20 (d8: 9.8 ± 0.6, d15: 7.6 ± 0.6, d20: 5.4 ± 0.5%, Figure 5B).

#### Activity

Activity was not affected by pre- or post-weaning treatment. The time spent standing and walking decreased over time, as it was higher on d8 than on d15 and 20, and it was higher on d15 than on d20 (d8: 3.4 ± 0.2, d15: 2.7 ± 0.2, d20: 1.7 ± 0.1%, Figure 5C). At the same time, the time spent inactive increased over time, as it was lower on d8 and 15 compared to d20 (d8: 57.8 ± 1.1, d15: 59.0 ± 1.2, d20: 61.8 ± 0.9%, Figure 5D).

#### Exploratory Behavior

Pre-weaning treatment did not affect exploratory behavior. Post-C piglets explored the environment more than Post-L piglets (Post-C: 16.6 ± 0.7, Post-L: 12.5 ± 0.5%). Overall, piglets spent more time exploring the environment on d8 than on d15 and 20 (d8: 17.4 ± 0.9, d15: 13.2 ± 0.7, d20: 13.0 ± 0.6%, Figure 5E). Post-L piglets spent more time exploring the experimental feeder (including tubes) than Post-C piglets (Post-C: 1.0 ± 0.1, Post-L: 4.0 ± 0.2%), and the time spent exploring the enrichment device was higher on d15 than on d8, with d20 in between (d8: 2.1 ± 0.3, d15: 2.8 ± 0.3%, Figure 5F).

#### Pig-Directed Behavior

Pig-directed behavior was not influenced by pre-weaning treatment. Compared to Post-C piglets, Post-L piglets spent less time nosing pen mates (Post-C: 2.6 ± 0.1, Post-L: 1.9 ± 0.1%). Also, less time was spent on nosing pen mates on d8 than on d15 and 20 (d8: 1.6 ± 0.1, d15: 2.7 ± 0.2, d20: 2.4 ± 0.3%, Figure 5G). The time spent on oral manipulation of pen mates was affected by post-weaning treatment, day, and their two-way interaction. Post-hoc analysis indicated that only on d8 Post-C piglets spent more time performing this behavior than Post-L piglets (Post-C: 2.1 ± 0.3, Post-L: 0.8 ± 0.2%). Post-C piglets did not differ in their time spent on oral manipulation of pen mates over time, while Post-L piglets spent less time on oral manipulation of pen mates on d8 compared to d15 and 20 (d8: 0.8 ± 0.2, d15 2.3 ± 0.3, d20: 2.2 ± 0.2%, Figure 5H).

#### Play Behavior

The time spent playing was affected by post-weaning treatment, day, and their two-way interaction, but not by pre-weaning treatment. Post-C piglets spent more time playing than Post-L piglets on d8 (Post-C: 2.1 ± 0.3, Post-L: 1.1 ± 0.1%) and on d15 (Post-C: 2.7 ± 0.2, Post-L: 1.8 ± 0.2%), and they spent more time playing on d15 than on d20 (d15: 2.7 ± 0.2, d20: 1.8 ± 0.1%). Post-L piglets spent more time playing on d15 and 20 than on d8 (d8: 1.1 ± 0.1, d15: 1.8 ± 0.2, d20: 2.0 ± 0.2%, Figure 5I).

### Affective State

#### Novel Environment Test

The first factor obtained from the factor analysis on the NET variables had high positive loadings for the time spent moving and the vocalizing frequency, and high negative loadings for the time spent exploring the environment and the latency to explore the feed bowl. The second factor had high positive loadings for the time spent standing and standing alert, and high negative loadings for the time spent exploring the feed bowl (Table 4). Pre-weaning treatment, post-weaning treatment and their interaction did not affect the piglets' scores on both factors (Factor 1: CC−0.09 ± 0.18, CL 0.23 ± 0.14, LC 0.06 ± 0.18, LL−0.19 ± 0.29, pre-weaning treatment: *p* = 0.505, post-weaning treatment: p=0.864, interaction: *p* = 0.157; Factor 2: CC 0.06 ± 0.12, CL 0.14 ± 0.23, LC−0.04 ± 0.23, LL−0.16 ± 0.19, pre-weaning treatment: *p* = 0.324, post-weaning treatment: *p* = 0.946, interaction: *p* = 0.621).

#### Attention Bias Test

Comparing behavior of piglets that did or did not receive a threat during the ABT showed that piglets receiving a threat spent less time moving and tended to spend more time standing alert. No other behaviors were affected by the threat (Table 8). During the threat, Pre-L piglets paid more attention to the threat than Pre-C piglets. No other behaviors performed during the threat were affected by pre-weaning treatment, post-weaning treatment, or their interaction (Table 9).

**Table 8:** Behavior performed in the attention bias test with or without receiving a threat.

**Behavior**	**With threat**	**Without threat**	***P*-value**
Moving (% of time)	24.0 ± 1.5	29.5 ± 2.1	**0.047**
Standing (% of time)	2.5 ± 0.4	3.3 ± 0.6	0.291
Standing alert (% of time)	48.5 ± 2.6	39.7 ± 3.5	*0.054*
Exploring environment (% of time)	20.5 ± 1.7	22.3 ± 3.0	0.538
Exploring feed bowl (% of time)	4.4 ± 0.8	5.2 ± 1.5	0.603
Latency exploring feed bowl (s)	73.1 ± 9.6	56.8 ± 12.4	0.343
Eliminating (frequency)	0.5 ± 0.1	0.4 ± 0.2	0.327
Vocalizing (frequency)	46.2 ± 4.1	55.9 ± 7.0	0.204

*Significant effects (p < 0.05) are indicated in bold and trends (p < 0.1) are indicated in italic. Data are presented as pen means ± SEM*.

**Table 9:** Behavior performed in the attention bias test during the 10 s threat.

**Behavior**	**Pre-C**		**Pre-L**		***P*** **-value**		
	**Post-C**	**Post-L**	**Post-C**	**Post-L**	**Pre**	**Post**	Pre^*^Post
Attention to threat (% of time)	50.0 ± 5.2	50.2 ± 6.5	67.2 ± 4.2	66.3 ± 5.4	**0.008**	0.760	0.994
Moving (% of time)	45.6 ± 5.5	43.4 ± 7.1	48.7 ± 7.2	44.4 ± 5.5	0.757	0.387	0.992
Standing alert (% of time)	32.7 ± 6.2	47.3 ± 7.0	39.3 ± 7.7	40.1 ± 6.6	0.987	0.246	0.321
Exploring environment (% of time)	13.4 ± 5.6	5.1 ± 2.1	7.4 ± 2.0	6.7 ± 2.1	0.664	0.178	0.284
Exploring feed bowl (% of time)	6.5 ± 3.7	2.9 ± 1.6	2.9 ± 1.9	5.5 ± 3.4	0.897	0.935	0.283
Vocalizing (frequency)	0.6 ± 0.2	1.2 ± 0.5	0.25 ± 0.2	0.8 ± 0.4	0.436	0.296	0.417

Behavior performed during the 10 s threat of pigs that had access to either only creep feed (Pre-C) or creep feed and live black soldier fly larvae (Pre-L) pre-weaning, and consequently had access to no larvae (Post-C) or had access to live black soldier fly larvae (Post-L) post-weaning. P-values of the effect of pre-weaning treatment (Pre), post-weaning treatment (Post) and their interaction (Pre^*^Post) are presented, and significant effects (p < 0.05) are indicated in bold. Data are presented as pen means ± SEM.

The first factor obtained from the factor analysis on piglet behavior in the ABT during the 150 s after the threat had high positive loadings for the time spent moving, time spent exploring the feed bowl, and vocalizing frequency, and high negative loadings for the time spent paying attention to the threat location, time spent standing alert and latency to explore the feed bowl. The second factor had a high positive loading for time spent exploring the environment and a high negative loading for time spent alert. The third factor had a high positive loading for the time spent standing and the eliminating frequency (Table 4). The pre-weaning treatment, post-weaning treatment, and their interaction did not affect the piglets' scores on any of the factors (Factor 1: CC 0.11 ± 0.27, CL−0.11 ± 0.17, LC 0.40 ± 0.24, LL−0.28 ± 0.26, pre-weaning treatment: *p* = 0.781, post-weaning treatment: *p* = 0.195, interaction: *p* = 0.511; Factor 2: CC−0.27 ± 0.37, CL−0.02 ± 0.16, LC−0.03 ± 0.13, LL 0.17 ± 0.26, pre-weaning treatment: *p* = 0.366, post-weaning treatment: *p* = 0.229, interaction: *p* = 0.915; Factor 3: CC−0.24 ± 0.17, CL 0.10 ± 0.32, LC 0.32 ± 0.27, LL−0.08 ± 0.28, pre-weaning treatment: *p* = 0.428, post-weaning treatment: *p* = 0.976, interaction: *p* = 0.111).

## Discussion

We investigated the effect of providing piglets with live black soldier fly larvae (BSFL) as edible enrichment during the pre- and/or 3 weeks post-weaning period. Pre-weaning larvae provisioning did not improve feed intake and body weight gain and had no effect on the indicators of affective state. However, it did lead to several changes in gastro-intestinal tract development around weaning. Post-weaning larvae provisioning reduced post-weaning feed intake but not total body weight gain, and it reduced oral manipulation of pen fixtures and pen mates.

### Pre-weaning

Larvae provisioning from d3 after birth until weaning did not influence the total time spent on eating, the number of piglets eating, and the distribution of total (creep feed and/or larvae) eater types. Previously, it was found that providing a diverse diet (including creep feed, celery, cereal honey loops, and peanuts in shell) improved overall feed intake and time spent eating compared to providing only creep feed (23), and providing creep feed that varied daily in flavor (including various fruity and sweet flavors) increased feed intake and feeder visits compared to providing creep feed with a uniform flavor (25). In these studies, the degree of dietary diversity was relatively high, as, respectively, four different feed items and five different flavors were used, compared to two feed items applied in the current study. Under natural conditions, young piglets also sample a large variety of feed items [reviewed by Ballari and Barrios-García (55)], and a higher degree of dietary diversity may be required to improve pre-weaning feed intake. In line with this, we see that piglets that received larvae before weaning (Pre-L piglets) spent a similar amount of time eating as piglets provided with two types of creep feed simultaneously (24), while their time spent eating was substantially lower than that of piglets provided with four feed items simultaneously (23) in studies with a similar set-up and with similar observation periods as the current study. As such, providing only larvae in addition to creep feed was not sufficient to increase the time spent eating before weaning.

Furthermore, the absence of any effect of pre-weaning larvae provisioning on total eating behavior may in part be due to the relatively high percentage of control (Pre-C) piglets that had consumed creep feed at weaning. Almost 100% of Pre-C piglets had sampled creep feed at weaning, which is higher than previously reported [e.g., ± 34% (56), ± 48% (57), ± 70% (28) at weaning on d28]. The relatively high percentage of eaters per litter can be a function of multiple factors that were previously found to affect (creep) feed intake, such as large pen size (58), numerous feeding spaces (59–61), early feed provisioning (62), higher weaning age (63), and/or creep feed composition (63–65). The large amount of time spent eating of Pre-C piglets may have abated any beneficial effects of larvae provisioning.

While total eating behavior did not differ, the time spent eating creep feed and the percentage of creep feed eaters per litter were negatively affected by larvae provisioning, particularly close to weaning. Consequently, the distribution of creep feed eater types was more skewed toward worse creep feed eaters in the Pre-L compared to the Pre-C treatment. Previously, providing a diverse diet also decreased consumption of creep feed compared to when only creep feed was provided (23). In that study, piglets preferred exploring the other feed items, and this was correlated to an increased time spent eating these feed items. Other studies also indicated that facilitating exploration, for example by means of a play feeder, draws more piglets to the feeder (66) and can increase feed consumption (67). Indeed, exploring feed items, or “foraging,” is often a precursor for feed intake (19). In the current study, interacting with larvae was also preferred over interacting with creep feed by Pre-L piglets, and Pre-L piglets generally spent more time exploring feed than Pre-C piglets. Larvae presumably facilitate exploration due to their preferred nutritional (high fat/protein) and textural (high moisture content) attributes (42), and this coincides with an increased time spent eating larvae at the expense of creep feed. Actual consumption of feed items could not be measured in the current study, so the effects on that are unknown. Despite the preference for larvae, of all Pre-L piglets that sampled solid feed before weaning approximately 94% (based on behavioral observations) sampled both creep feed and larvae at least once.

Because creep feed and BSFL have a different nutritional composition, and Pre-C and Pre-L piglets differed in their time spent eating creep feed and larvae, treatment may have influenced nutrient intake. However, pre-weaning piglet growth and weaning weight were not influenced by treatment. Any potential effects on growth caused by differences in nutrient uptake from the provided feed items were likely overruled by the uptake of nutrients from the sow's milk, as this is the piglets' main nutrient source during lactation.

### Post-weaning

Some gastrointestinal tract measures on d3 post-weaning were affected by larvae provisioning. First, the proximal stomach digesta of piglets provided with larvae around weaning (LL piglets) had a higher pH than that of piglets without larvae (CC piglets). The proximal region of the stomach acts as a feed reservoir (68). LL piglets consumed whole larvae that are more rigid than feed pellets and may also be less easily digested because chitin in the larvae integument can hinder protein digestion (69). These larvae may have hindered mixing of gastric juices with feed in the proximal stomach. This was also found in previous studies comparing rigid (e.g., roasted almonds) to soft (e.g., cooked rice) feed items (70, 71). The consequences of a higher proximal stomach digesta pH for nutrient utilization are unclear, as this depends on numerous other factors such as gastric emptying rate and buffering capacity (70, 71).

The caecum of LL piglets was longer and marginally heavier than that of CC piglets. This could also be attributed to the larvae's larger size and potentially lower digestibility compared to creep feed. If certain feedstuffs cannot be digested in the small intestine, a larger volume of undigested feed will reach the large intestine (72, 73). This can affect large intestine development, for example by increasing the size and/or weight of the cecum and colon (30, 72, 73). Larvae consumption could have similarly increased cecum fill and thereby promoted cecum growth. However, colon size and weight and digesta weight were not affected by larvae provisioning, and the exact mechanisms causing the observed effect require further investigation.

While colon size was not affected by treatment, passage of FITC through the colon wall was lower in LL piglets compared to CC piglets, though TRITC passage was only numerically reduced. It is expected that the smaller FITC molecule (4 kD) is transported paracellularly, while the larger TRITC molecule (40 kD) is likely transported both trans- and paracellularly. Thus, only paracellular transport seems to be higher in CC piglets, and this can increase the chance of pathogens crossing the intestinal epithelium [as reviewed by Pluske et al. (15), Modina et al. (74), and Wijtten et al. (75)], posing a health risk. As suggested above, the undigested fraction of larvae may have increased the large intestinal fill in LL piglet. Increasing large intestinal fill was previously found to benefit colonic intestinal barrier function by changing the microbiota composition (76) or by promoting epithelial cell differentiation (77), and the undigested fraction of larvae may have had a similar effect.

Finally, LL piglets had significantly less glucose passage through the colon wall and numerically less glucose passage through the jejunum wall than CC piglets. Intestinal glucose absorption occurs through active NA^+^ dependent transport, which happens mainly in the small intestine (78). CC piglets spent more time eating creep feed while LL piglets spent more time eating BSFL, and because creep feed has a much higher carbohydrate level than BSFL, the carbohydrate intake and intestinal carbohydrate level of CC piglets was likely higher. Intestinal carbohydrate levels are positively linked to the expression of glucose transporters in the intestine (79), therefore LL piglets may have had lower intestinal glucose transporter expression, possibly explaining the lower glucose passage across the intestinal wall. Around weaning, decreased glucose passage could be an indication of impaired intestinal barrier function (74), however this is not supported by the observed FITC and TRITC passage rates.

Overall, the presence or absence of live BSFL around weaning had diverging effects on GIT development and functioning, and most observed differences were minor. Accordingly, pre- and post-weaning BSFL provisioning had no effect on the number of days piglets had (watery) diarrhea. Post-weaning diarrhea is a multi-factorial problem [reviewed by Heo et al. (14)], and feed intake has had contrasting effects on diarrhea occurrence (8, 23, 64, 80). Including BSFL fat in weanling piglets' diet also did not affect diarrhea rate during 4 weeks post-weaning (45). More research is required to determine the exact mechanisms by which live BSFL consumption affects GIT development, intestinal permeability, and diarrhea occurrence.

Pre-weaning treatment influenced post-weaning performance. Pre-C piglets tended to grow faster during d0-1, while Pre-L piglets tended to grow faster during d2-7 post-weaning, irrespective of feed intake. Significant differences only occurred during d14-21 post-weaning, where Pre-C piglets ate more and grew faster than Pre-L piglets. The timing of these effects contrasts with several other studies that found benefits of increased pre-weaning feed intake mainly directly after weaning (6, 27, 81, 82), often followed by a reduction in treatment differences over time (27, 81, 82). The inconsistent results directly after weaning may be due to the confounding effect of post-weaning treatment on performance. Also, there was a high variation in growth rate between pens directly after weaning. During week 3 post-weaning piglet performance became less variable, and an improved performance of Pre-C piglets became apparent. However, body weight on d21 was not affected by pre-weaning treatment, likely because of the variability in growth rate and feed intake throughout the 3 weeks post-weaning. The results suggest that pre-weaning larvae provisioning does not aid the dietary transition at weaning as opposed to providing only creep feed. Even though increased feed exploration pre-weaning was previously found to benefit post-weaning feed intake (66), in the current study the effects of increased exploration facilitated by larvae were likely superseded by the high time spent eating creep feed of Pre-C piglets. Creep feed is more similar in texture and nutritional composition to the weaner diet than larvae, and familiarity with these features presumably eased the weaning transition (47) and improved post-weaning feed intake and growth in Pre-C piglets. Similarly, familiarity with larvae before weaning also marginally increased larvae intake after weaning. Due to the relatively low intake of larvae compared to weaner feed, this did not affect post-weaning piglet performance.

Compared to the pre-weaning treatment, the post-weaning treatment had a higher impact on post-weaning performance, as the time spent eating and the pellet and total dry matter intake were continuously higher for Post-C piglets than for Post-L piglets. These results contradict with other studies where enrichment such as extra space, straw and/or peat improved piglet feed intake (32, 52). In contrast to straw and peat, larvae have a high nutritional value and were therefore expected to have a different impact on feed intake and performance. Larvae are very palatable due to their high levels of fat and protein (38), and short preference tests indicated that eating larvae is preferred over eating regular feed pellets (42). In line with this, both the current and a previous study observed high levels of interaction with larvae, and a simultaneous reduction in time spent eating feed (41). The high intake of larvae likely increased the feeling of satiety (83), and as such it reduced the motivation to eat pellets and subsequently lowered total feed consumption. Additionally, the consumption rate of larvae seems to be slower than that of pellets, as Post-L piglets spent equal amounts of time eating larvae and pellets, but the dry matter intake of larvae was substantially lower. Therefore, larvae may satisfy the exploratory and eating motivation of piglets at lower intake levels than feed, resulting in a lower overall feed intake. It must be noted that, despite the increased feed intake, Post-C piglets only experienced a temporary higher growth rate, and body weight on d21 post-weaning was not affected by post-weaning treatment. This indicates that Post-L piglets maintained a similar growth rate as Post-C piglets despite the lower feed intake, and suggests that Post-L piglets may have been more efficient in their feed conversion, though this must be confirmed in future studies.

Concerning behavior, pre-weaning treatment only influenced the time spent eating, whereas post-weaning treatment influenced a range of behaviors. The larger effect on behavior of the current environment as opposed to the former environment was expected as the presence of larvae mainly influences these behaviors, and this was also observed previously for numerous enrichment items (18, 33). Post-C piglets spent more time on exploring the environment and nosing pen mates on all observation days, and they spent more time on manipulating pen mates and playing on some of the observation days. On the other hand, Post-L piglets continuously spent more time on exploring the enrichment device and eating larvae. Overall, larvae provisioning clearly facilitated exploratory behaviors, redirecting exploration away from the pen and pen mates. These results are similar to a study in which small amounts of larvae were provided for 11 days post-weaning (41). Exploring larvae is likely more satisfying than exploring pen fixtures or other pigs, as larvae have more characteristics that are preferred by pigs, such as being odorous, destructible and edible (84). Furthermore, pig-directed oral manipulation has been associated with a higher presence of painful lesions and wounds (85, 86), therefore redirecting exploration away from pigs by providing larvae can benefit piglet welfare.

Some effects of larvae provisioning on behavior varied over time. Post-C piglets spent more time manipulating pen mates and playing than Post-L piglets only on d8 and d8 and 15 post-weaning, respectively. For Post-L piglets, the time spent eating larvae decreased on d15 and 20 compared to d8, and concurrently the time spent manipulating pen mates and playing increased over time. Therefore, it seems piglets redirected their activity away from larvae and toward their pen mates later in the post-weaning period, explaining the absence of treatment effects during those days. It is unlikely that piglets lost interest in the larvae over time, as most pens consumed the maximum amount of larvae every day in week 3 post-weaning. A more likely explanation is that piglets became more efficient over time in retrieving the larvae from the tubes, as this was also observed in a previous study where piglets had access to tubes containing larvae (42). In the current study, larvae were provided at the same time every day, and this temporal predictability may have exacerbated beneficial effects on behavior and welfare (87, 88), though it may also have diminished interaction with the enrichment device containing larvae in-between provisioning moments. Prolonging the engagement with larvae may require changes in the amount, manner and/or timing of larvae provisioning.

Piglets that received a threat during the Attention Bias Test (ABT) spent less time moving and more time standing alert than piglets that did not receive a threat. Reduced locomotion and increased vigilance have previously been observed in piglets in response to a novel stimulus and have been linked to increased fearfulness (89–91), suggesting that the negative stimulus used in the ABT was considered a threat. Contrarily, the applied positive stimulus, namely a feed bowl filled with feed pellets mixed with corn and raisins, did not receive as much attention from the pigs compared to a previous study where feed mixed with chocolate peanuts and carrots was provided in an ABT (52). This may be a result of the generally low and variable feed intake recently weaned piglets (92), causing these piglets to not yet be habituated to the feed and not consider it a positive stimulus. To improve the design of the ABT for recently weaned piglets, providing a different positive stimulus may be required.

Both factors retrieved from the factor analysis on the Novel Environment Test (NET) responses included behaviors that have previously been linked to fearfulness, such as low exploration of the environment and a high frequency of vocalizations in Factor 1, and a high time standing alert in Factor 2 (90, 93). Pre- and post-weaning treatment did not influence piglets' responses during the NET, therefore we have no indication that larvae provisioning affected piglets' fearfulness. Environmental enrichment found to decrease piglet fearfulness includes marginally increased space and more toys (94), hanging ropes and tires (95), and live BSFL provided during 11 days after weaning (41). Compared to this last study that also included larvae provisioning, the NET in the current study was performed closer to weaning (d4 instead of d10-11 post-weaning), therefore there was less time for post-weaning larvae provisioning to impact piglet fearfulness, possibly explaining the contradictory results. Pre-L piglets did have extensive experience with larvae provisioning before weaning, however pre-weaning treatment also did not affect NET responses. Previously, pre-weaning dietary diversity also did not influence NET responses at weaning (96). It appears that increasing dietary diversity by providing creep feed and larvae before weaning does not habituate piglets more to novelty than providing only creep feed in the current experimental setting. Under more barren commercial conditions, effects may differ.

Factor 1 retrieved from the ABT reflects the direction of attention bias of the piglets, where positive scores on this factor relate to an attention bias toward the positive stimulus (the feed bowl) and away from the negative stimulus (the threat location). Larvae provisioning before or after weaning did not affect the piglets' scores on this factor, therefore larvae provisioning did not result in a more positive or negative attention bias, reflective of an animal's affective state (97, 98). Previously, enrichment had a positive (35) or no (52, 99) effect on pig's affective state. As mentioned before, the positive reward provided in the ABT may not have been viewed as positive by all pigs, therefore any bias in attention may not have been related to the positive perception of feed, but more to exploration in general. Also, as observed in the NET, larvae provisioning may not have been sufficient to improve the affective state of newly weaned piglets. Pre-L piglets did pay more attention to the threat during the 10 s the threat was present than Pre-C piglets. Previous studies have associated increased attention toward a threat with either increased (52) or reduced (100) anxiousness. As overall responses to the NET and ABT did not differ, it is possible that the increased attention was not linked to the piglet's affective state. Instead, the increased interest in the threat may be due to a more positive association with a disturbance, caused by regularly receiving more preferred larvae as opposed to only less preferred feed pellets (42) before weaning. As these results are based on a 10 second period, they should be interpreted with caution.

In conclusion, pre-weaning larvae provisioning increased feed-directed exploration, decreased the time spent eating creep feed, and did not affect the overall time spent eating feed before weaning. Continuous larvae provisioning around weaning affected cecal and colonic development and proximal stomach digesta pH. After weaning, larvae provisioning redirected exploration away from pen fixtures and pen mates and toward the larvae. Larvae provisioning also reduced post-weaning feed intake without affecting piglet growth rate and body weight on d21 post-weaning. Affective state assessed in behavioral tests shortly after weaning were not influenced by larvae provisioning. Overall, larvae were easily accepted from a young age onwards, yet they did not have a large impact on the weaning transition. In the current set-up larvae provisioning was more beneficial for piglet welfare post-weaning compared to piglet feed intake pre-weaning. However, the impaired post-weaning feed intake that accompanied larvae provisioning indicates that a different method or amount of larvae provisioning may be more appropriate to support piglet welfare.

## Data Availability Statement

The raw data supporting the conclusions of this article will be made available by the authors, without undue reservation.

## Ethics Statement

The animal study was reviewed and approved by the Animal Care and Use Committee, Wageningen University and Research.

## Author Contributions

AI, WG, EB, BK, and JB designed the experiment. AI, MM, and BL conducted the experiment. AI analyzed the data, wrote the manuscript, and prepared the figures. WG and JB advised on data analysis. WG, EB, MM, BL, BK, and JB substantially revised the manuscript. All authors contributed to the article and approved the submitted version.

## Funding

The study was funded by the Netherlands Organization for Scientific Research (NWO) under project number 15567. It was co-funded by HatchTech, ForFarmers and Bestico.

## Conflict of Interest

The authors declare that the research was conducted in the absence of any commercial or financial relationships that could be construed as a potential conflict of interest.

## Publisher's Note

All claims expressed in this article are solely those of the authors and do not necessarily represent those of their affiliated organizations, or those of the publisher, the editors and the reviewers. Any product that may be evaluated in this article, or claim that may be made by its manufacturer, is not guaranteed or endorsed by the publisher.
