# Supplementary material for: Assessing the Effectiveness of Providing Live Black Soldier Fly Larvae (Hermetia illucens) to Ease the Weaning Transition of Piglets
Source: Front Vet Sci. 2022 Feb 16;9:838018. doi: 10.3389/fvets.2022.838018 (PMC8890697; doi:10.3389/fvets.2022.838018)
Supplement: Supplementary file 1 [file Data_Sheet_1.PDF]

Supplementary Table S1. Nutrient composition of creep feed

Calculated nutrient composition[1]	Creep feed
Net energy	1010
Dry matter	872
Crude protein	208
Crude fat	45
Crude ash	61
Crude fiber	43
Starch	497
Sugars	41
Non-starch polysaccharides[2]	148
Calcium	8.6
Phosphorus	6.5
Magnesium	1.8
Potassium	8.5
Sodium	1.8
Chloride	4.5
Standard ileal digestible lysine	12
Standard ileal digestible methionine	4.5
Standard ileal digestible threonine	7.1
Standard ileal digestible tryptophan	2.3

[1]Calculated nutrient composition according to CVB (2016). Nutrients are presented in g/kg dry matter, except net energy (kJ/100 g) and dry matter (g/kg).

[2]Calculates on a dry matter basis as = 1000-crude protein-crude fat-crude ash-starch-sugars.

Reference

CVB. (2016). *Veevoedertabel 2016: chemische samenstelling en nutritionele waarden van voedermiddelen*. Den Haag, The Netherlands: Centraal Veevoederbureau.

Supplementary Table S2. Ingredient composition of creep feed

Ingredient composition[1]	%
Wheat	27.1
Barley	25
Maize	20
Soybean meal	16.5
Sunflower meal	6
Soybean oil	1.3
Vitamin and mineral premix	0.5
Limestone fine	1.3
Monocalcium phosphatase	0.9
Salt	0.4
Citric acid	0.1
L-lysine hydrochloride	0.52
DL-methionine	0.15
L-threonine	0.16
L-tryptophan	0.05
L-valine	0.02

[1]Feed colorant Indigo carmine was included in the feed at 5 g/kg feed.
